# Collective outbreak of severe acute histoplasmosis in immunocompetent Chinese in South America: the clinical characteristics and continuous monitoring of serum cytokines/chemokines

**DOI:** 10.1186/s12875-022-01771-2

**Published:** 2022-08-08

**Authors:** Yin-yin Peng, Shu-liang Guo, Xiao-feng Yan, Lv-lang Zhang, Jing Wang, Guo-dan Yuan, Gang Qing, Lu-lu Xu, Qian Zhan

**Affiliations:** 1 Department of Respiratory and Critical Care Medicine, The First Affiliated Hospital of Chongqing Medical University, Chongqing, 400016 China; 2 Department of Hematology Medicine, The First Affiliated Hospital of Chongqing Medical University, Chongqing, 400016 China; 3 Chongqing Public Health Medical Center, Chongqing, 400016 China; 4 The Center for Clinical Molecular Medical detection, The First Affiliated Hospital of Chongqing Medical University, Chongqing, 400016 China

**Keywords:** Histoplasmosis|, Pulmonary, Infection, Fungi, Outbreak, Cytokines

## Abstract

**Background:**

Acute histoplasmosis is a rare fungal disease in China. This study is aimed to summarize the clinical characteristics of the first large-scale outbreak of imported acute histoplasmosis in Chinese, so as to provide suggestions for clinical diagnosis and treatment.

**Methods:**

We collected the symptoms, signs, laboratory examination and imaging data of 10 patients in so far the biggest outbreak of imported acute histoplasmosis in immunocompetent Chinese. Their clinical characteristics and time-varying cytokine/chemokine levels were analyzed, and rank correlation analysis between these markers was utilized to show their condition.

**Results:**

The 10 patients of imported acute histoplasmosis were working without any respiratory protection in an abandoned mine tunnel in Guyana. The most common symptoms were fever and cough. Their chest CT imaging showed multiple nodular shadows in lungs. Laboratory examination showed that at admission the CRP, PCT, LDH, CysC, G-test, β2-MG were all increased in at least 9 patients, and the CD4/CD8 was decreased to < 1 in all patients. Most cytokines/chemokines (other than IL-4, IL-12, INF-α, TNF-α) varied widely with patients and time, but their overall trend is higher at admission and decreasing gradually during hospitalization, especially for the IL-6, IL-8, IL-10 and IFN-γ. The LDH, CysC, G-test, β2-MG, N/L, IL-6, IL-8, IL-10, IFN-γ, IL-27 are in positive associations to both CRP and PCT.

**Conclusions:**

The diagnosis of acute histoplasmosis needs a comprehensive analysis of epidemiological history, clinical symptoms and signs, and results of imaging, laboratory, microbiological and pathological examinations. Although none of the CRP, PCT, G-test, N/L, LDH, CysC, β2-MG, IL-6, IL-8, IL-10, IFN-γ shows specificity in the diagnosis of acute histoplasmosis, there is possibility that the above factors might help in the inflammation and prognosis estimation. However, more studies and further investigation are still required for the verification.

**Supplementary Information:**

The online version contains supplementary material available at 10.1186/s12875-022-01771-2.

## Background

Histoplasma capsulatum (H. capsulatum), the causative agent to histoplasmosis, is found in association with birds, bats, or their droppings. Exposure to H. capsulatum typically occurs by inhalation of fungal spores, following disruption of soil or other contaminated material for histoplasma’s habitats [1]. H. capsulatum is a thermally dimorphic fungus, and the average incubation period is 1 ~ 3 weeks.

Histoplasmosis is a rare tropical endemic and fungal infectious disease. According to the time of onset, it is divided into acute (< 1 month), subacute (1 ~ 3 months), and chronic (> 3 months). Its clinical manifestations can range from mild asymptomatic infection to severe life-threatening disease, depending on host status, inoculum size, and other factors [1]. Acute pulmonary histoplasmosis is the most common symptomatic manifestation, and is often self-limited, especially among immunocompetent persons [2].

Histoplasmosis is endemic in the Mississippi and Ohio river basins of United States, Latin America, Africa, and part of Asia [3]. In the endemic areas, the diagnostic methods of histoplasmosis include culture, pathological diagnosis, and antigen/antibody tests. Especially the H. capsulatum antigen has become a powerful tool for rapid detection of acute histoplasmosis [4–6]. Besides, novel diagnostic methods such as polymerase chain reaction (PCR), or Metagenomic Next-generation Sequencing (mNGS) can also be utilized, but those methods are generally more expensive.

In China, histoplasmosis is more rare, sporadic, and chronic, and the H. capsulatum antigen is commonly unavailable. Thus the diagnosis of histoplasmosis in China usually relies on ordinary methods like culture or pathological diagnosis, but without the aid of H. capsulatum antigen. In China the histoplasmosis is hard to be rapidly distinguished from tuberculosis, pulmonary metastasis tumour, or other pulmonary fungal infections in clinic.

For improving the diagnosis of histoplasmosis in non-endemic countries like China, a reasonable option is to find other obtainable markers. For example, as in histoplasmosis patients the alkaline phosphatase, Westergren sedimentation rate, lactate dehydrogenase (LDH), C-reactive protein (CRP), ferritin expression, and fungal (1–3)-β-Dglucan are all elevated [6, 7], However, none of them shows specificity in the diagnosis of acute histoplasmosis.

In this study, we collected data during hospitalization of an imported acute histoplasmosis outbreak, in which 10 Chinese were infected in South America. So far, this outbreak is still the biggest collective outbreak in immunocompetent Chinese. We 1) collected the symptoms, signs, laboratory examination and imaging data of the 10 patients; 2) summarized and analyzed their clinical characteristics and their time-varying cytokine/chemokine levels; and 3) performed a Spearman’s rank correlation analysis to the laboratory test markers, in order to share our experiences and lessons about the acute histoplasmosis, provide clinicians better insights into this disease.

## Patients and methods

### Patients

The 10 patients were sent as miners from Chongqing to Guyana in South America, where they got infected. They returned to China on April 10th 2019 in Beijing, where their blood samples were taken for mNGS test by Chinese Center for Disease Control and Prevention. They were then transferred immediately and admitted into Chongqing Public Health Medical Center.

### Imaging examinations and laboratory tests

Imaging examinations and laboratory tests were performed immediately at the admission of patients. After that, computer tomography (CT) scanning was performed twice in the 1st week, and once from the 2nd week till discharge. Blood test was performed everyday in the 1st week, every other day in the 2nd week, 2 or 3 times in the 3rd week, and 1 or 2 times per week from the 4th week till discharge. The imaging examinations include CT scannings of their heads, chests and abdomens. The laboratory tests include the tests of blood routine, liver and kidney functions, CRP, procalcitonin (PCT), fungal (1–3)-β-Dglucan G-test (G-test), CD4/CD8, bronchoscopic frozen lung biopsy, as well as cultures of their blood, sputum, bone marrow, and bronchoalveolar lavage fluid (BALF) samples (Table 1).

**Table 1:** Examinations and laboratory tests to the 10 patients

patient No.	1	2	3	4	5	6	7	8	9	10
CT scannings of head, chest, and abdomen	yes	yes	yes	yes	yes	yes	yes	yes	yes	yes
blood routine, liver and kidney function, CRP, PCT, G-test, CD4/CD8	yes	yes	yes	yes	yes	yes	yes	yes	yes	yes
blood and sputum sample culture	yes	yes	yes	yes	yes	yes	yes	yes	yes	yes
bone marrow smear examination	yes	yes	yes	no	yes	yes	no	no	no	yes
BALF culture	yes	no	no	no	no	no	no	no	no	no
bronchoscopic frozen lung biopsy	yes	no	no	no	no	no	no	no	no	no

### Diagnosis

**Fig. 1:**
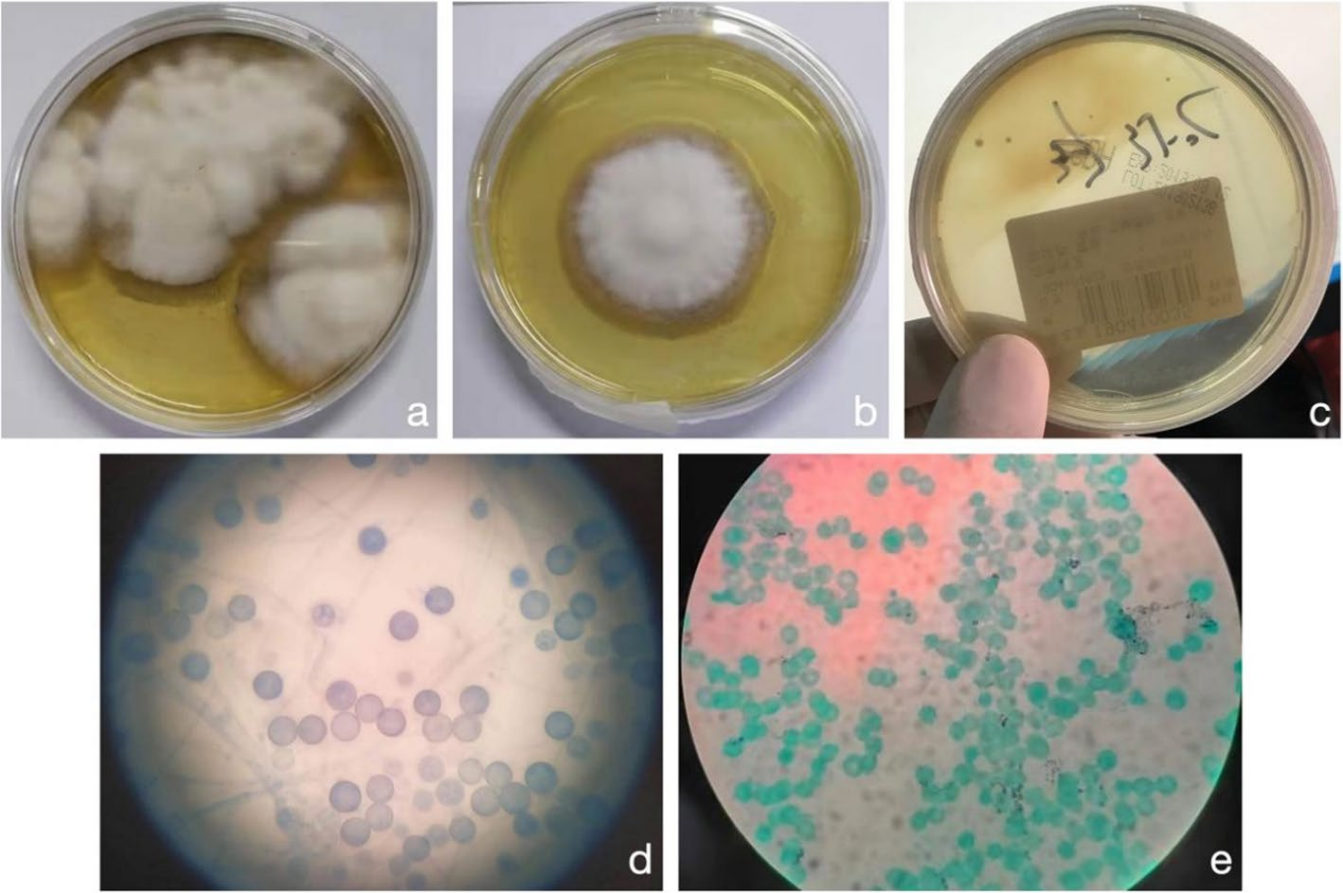
Culture and microscopy of bronchial lavage fluid (BALF) and blood samples from No.1 patient. **a** The culture of the BALF sample for the No. 1 patient showed that the colonies were grown into white cotton-like under 25°C. **b** The culture of the blood sample for the No. 1 patient showed that the colonies were grown into white cotton-like under 25°C. **c** [10] The culture of the BALF and blood sample for the No. 1 patient showed that the colonies were grown into smooth and cheese-like under 37°C. **d** The spores were round and thick walled, in cotton blue staining under microscope with 10 × 1000 magnification and oil-immersion objective lens. **e** [10] The spores were stained black in hexamine silver staining under microscope with 10 × 100 magnification and oil-immersion objective lens

At admission, patients were all speculated with acute histoplasmosis according to the revised definition of invasive fungal disease [8], because of: 1) their inducements, epidemiological characteristics, and clinical/imaging manifestations; 2) the indication of H. capsulatum by mNGS test of their blood samples; 3) exclusion of other diseases including tuberculosis and pulmonary metastasis tumour. Thus we determined to treat them according to the guidelines for histoplasmosis patient management, and at the same time we took and cultured their samples for further definitive diagnosis evidence. Finally after 37 days, they were definitively diagnosed with acute histoplasmosis, because fungi growth was found in the BALF and blood cultures of No.1 patient (on Sabouraud medium, under 25 °C and 37 °C, respectively). Fungi grew into white cotton-like colonies under 25 °C, but into smooth and cheese-like colonies under 37 °C. Within colonies, H. capsulatum was identified by cotton blue and hexamine silver stainings at the blastospores (3-5 μm in diameter) [9, 10] (Fig. 1). Consistently, the frozen section of lung also suggested multifocal histiocytosis with granulomatous lesion and local tissue necrosis.

### Treatment and prognosis

Patients were treated following the 2007 edition of clinical practice guidelines for the management of patients with histoplasmosis by the Infectious Diseases Society of America [11]. When hospitalized, all patients were treated with liposomal amphotericin B, and then with itraconazole. The No.1 patient was in deep coma at admission, and was assessed vegetative by neurologists. The treatment of the No.1 patient lasted 2 months, although the lung lesions were improved significantly, unfortunately he was still in coma. He was then transferred to a local hospital for rehabilitation, and lost follow-up. The other nine patients all achieved remission with all symptoms disappeared after only 3 weeks’ treatment, and they were discharged 37, 40, 44, 36, 37, 37, 37, 36, 36 days after admission, respectively. After discharge, they still kept taking oral itraconazole and regular follow up..

### Measurement of serum cytokines/chemokines

For exploring more about markers of disease condition, whenever the laboratory tests in Table 1 were performed, additional ethylenediamine tetracetic acid (EDTA) blood samples of the patients were taken for cytokines/chemokines measurements. Samples were preserved in ice packs and transported immediately to a biosafety level 2 laboratory in the First Affliated Hospital of Chongqing Medical University. Serum was separated in centrifuge (2000 g for 10 min at 4 °C), and stored in 3 mL aliquots at − 80 °C. Inflammatory cytokines/chemokines were measured simultaneously in multiplex bead-based flow cytometry by human inflammatory cytokine/chemokine cytometric bead array (4-color FACSCalibur flow cytometer, BD Biosciences). Besides, serum concentrations of IL-27, IL-33, and high mobility group protein (HMGB1) were measured using an enzyme-linked immunosorbent assay (ELISA) kit (R&D Systems). Totally 15 serum cytokines/chemokines were measured: IL-1β, IL-2, IL-4, IL-5, IL-6, IL-8, IL-10, IL-12, IL-17, IL-27, IL-33, IFN-α, IFN-γ, TNF-α, and HMGB1.

### Relevance

We employed the blood test results of patients in the first 3 weeks of treatment for relevance analysis (Table 1). Laboratory markers in Table 1 were checked, and top 9 abnormal markers beyond normal ranges were picked up for relevance analysis: CRP, PCT, G-test, CD4/CD8, WBC, N/L, β2-MG, Cysc, LDH. Spearman’s rank correlation analysis was performed twice: 1) between any two of the 10 laboratory markers; and 2) between the 10 laboratory markers and the 15 serum cytokines/chemokines. Subjectively, rank correlation coefficient was categorized into three groups: weak association (− 0.3 ~ 0.3), moderate association (− 0.7 ~ − 0.3, 0.3 ~ 0.7), and strong association (− 1 ~ − 0.7, 0.7 ~ 1).

## Results

### General data

Patient ages range from 30 to 56 years (median: 45). No.7 patient used to have tuberculosis and gastroesophageal reflux (both already cured). None of them had experienced HIV infection, or long-term use of glucocorticoid and immunosuppressant. And at the time of outbreak, all patients did not have comorbidities that may affect the tested markers of this study.

Patients were exposed to bat excrement on the ground of an abandoned mine tunnel, where they worked without respiratory protection. Their jobs include soil cleaners (No.1-No.4 patients), flame cutters (No.7, No.10), dockers (No.5, No.9) and site supervisors (No.6, No.8). They entered the tunnel successively, and received various total lengths of exposure, which range from 0.2 (dockers) to 96 hours (soil cleaners). Patients got fever successively about 9–13 days after their last entrances into the tunnel (Table 2). Besides, the No.1 patient started to be in drowsiness after 4 days’ fever, and a few days later he underwent a septic shock together with an acute respiratory distress syndrome (ARDS) and a sudden cardiac arrest after that. His heartbeat recovered after 15 minutes’ cardiopulmonary resuscitation, but he fell into coma and kept in it since then. He then received endotracheal intubation, and was transferred back to China together with the other patients.

**Table 2:** General information, symptoms and signs, and imaging examinations of the 10 patients

patient No.	1	2	3	4	5	6	7	8	9	10
**General information**										
age (year)	42	50	50	48	40	56	43	47	32	30
incubation period (day)	11	9	11	13	12	12	11	10	11	12
onset day	2019-03-27	2019-03-23	2019-03-27	2019-03-29	2019-03-29	2019-03-29	2019-03-27	2019-03-29	2019-03-29	2019-04-02
job	soil cleaner	soil cleaner	soil cleaner	soil cleaner	docker	site supervisor	flame cutter	site supervisor	docker	flame cutter
exposure time (hour)	48	36	54	96	0.2	0.3	4	0.3	0.2	2
**symptoms and signs**										
fever	yes	yes	yes	yes	yes	yes	yes	yes	yes	yes
chills	coma	no	yes	no	no	no	no	yes	no	no
insomnia	coma	yes	no	yes	yes	yes	no	yes	yes	yes
dizzy	coma	no	yes	yes	yes	yes	no	yes	yes	yes
headache	coma	no	yes	no	yes	no	yes	yes	yes	yes
cough	coma	yes	yes	yes	yes	yes	yes	yes	yes	yes
sputum	coma	yes	yes	no	no	yes	yes	yes	no	no
dyspnea	coma	yes	yes	yes	yes	yes	no	no	no	no
thoracodynia	coma	no	no	no	yes	no	no	no	no	no
anorexia	coma	yes	no	no	yes	no	yes	yes	yes	yes
nausea	coma	no	no	no	no	no	no	yes	no	yes
vomit	coma	no	no	no	no	no	no	yes	no	yes
diarrhea	no	no	no	no	no	no	no	yes	no	no
lung moist rales	yes	yes	no	yes	no	no	no	no	no	no
rash	yes	no	no	yes	no	no	no	no	yes	no
**imaging findings**										
multiple nodular shadows in chests	yes	yes	yes	yes	yes	yes	yes	yes	yes	yes
consolidation shadows in chests	yes	yes	no	no	no	no	no	no	no	no
ground-glass opacity shadows in chests	yes	yes	no	yes	yes	yes	yes	yes	yes	yes
mediastinum and/or hilar lymphadenopathy	yes	yes	yes	yes	yes	yes	yes	yes	yes	yes
pleural effusion	yes	no	no	no	yes	yes	no	no	no	yes
pericardial effusion	yes	no	no	yes	no	yes	no	no	no	no
meningeal thickening	yes	no	no	no	no	no	no	no	no	no
hepatomegaly	no	no	no	no	yes	no	no	no	no	no
splenomegaly	no	yes	yes	yes	yes	yes	no	yes	yes	yes

### Symptoms and signs

The symptoms of systemic inflammation and disorders of nervous, respiratory, digestive systems are in Table 2. Fever, cough, dizziness, headache, insomnia are the most common symptoms, and skin rash on anterior chest wall, splenomegaly, moist rale of lung are the main signs. The fevers are either persistent (in 3 patients), or intermittent (in 7 patients). The body temperatures during fever range from 38 °C to 39.4 °C.

### Imaging manifestations

**Fig. 2:**
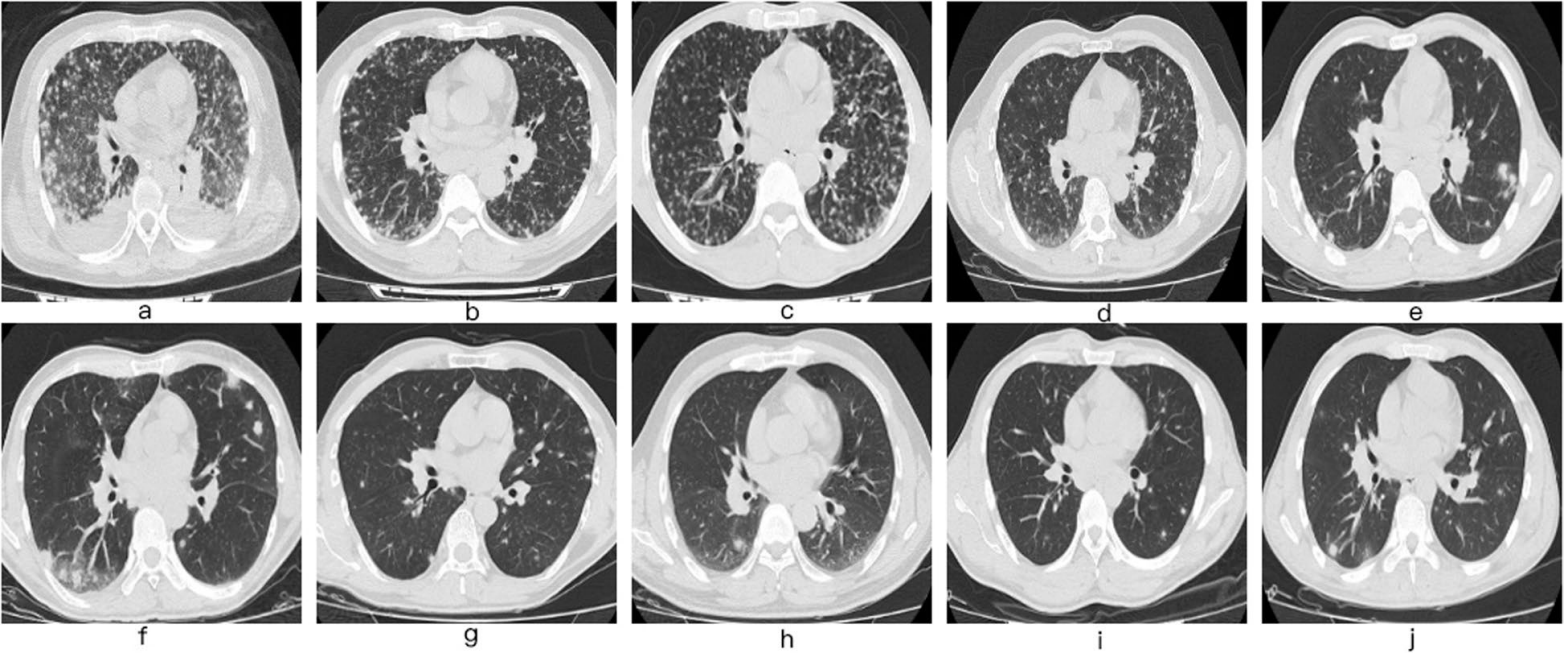
Manifestations of chest CT imaging of the 10 patients. The a-j refer to patients No.1-No.10 respectively

**Fig. 3:**
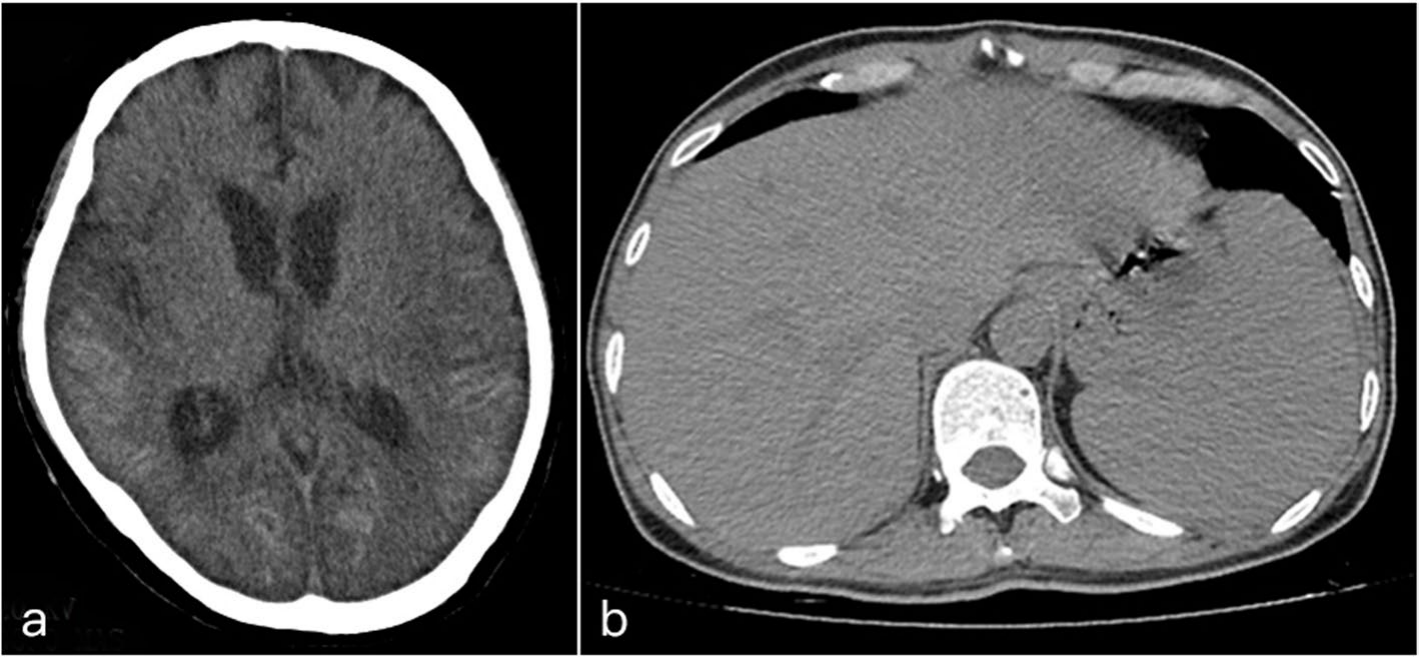
Manifestation of cranial CT imaging of the patient No.1 (**a**) [9], and manifestation of abdominal CT imaging of patient No.6 (**b**)

From CT scanning images the following were seen: 1) in chests: multiple nodular shadows, consolidation shadows, ground glass opacity shadows, mediastinum and/or hilar lymphadenopathy, pleural effusions, and pericardial effusions (Fig. 2); 2) in heads: significant increase in thickness and density of bilateral frontotemporal meninges and parietal/occipital lobes of No.1 patient (Fig. 3a) [9]; 3) in abdomens: splenomegaly (in 8 patients) and hepatomegaly (in 1 patient) (Fig. 3b). Details are shown in Table 2.

### Results of laboratory tests

At admission, except for No.6, other 9 patients had their PaO_2_/FiO_2_ all < 300 mmHg (supplement Table 1). The No.1 patient was assisted by invasive mechanical ventilator, and the others all used nasal cannulas or face masks for oxygen. The bone marrow examination of 6 patients (No. 1, 2, 3, 5, 6, 10) and the cerebrospinal fluid culture of No.1 patient all showed nothing abnormal.

The following laboratory markers were increased in all patients: CRP, PCT, LDH and CysC; the following were increased in 9 patients: G-test, β2-MG; the CD4/CD8 was decreased to < 1 in all patients; and the WBC was decreased in 1 patient. During treatment, the WBC occasionally increased beyond normal range in 3 patients. After treatment, as the symptoms remitted, the following markers gradually returned into normal ranges: CRP, PCT, LDH, β2-MG, G-test, WBC and CD4/CD8; but CysC still fluctuated.

### Results of serum cytokine and chemokine concentrations

**Fig. 4:**
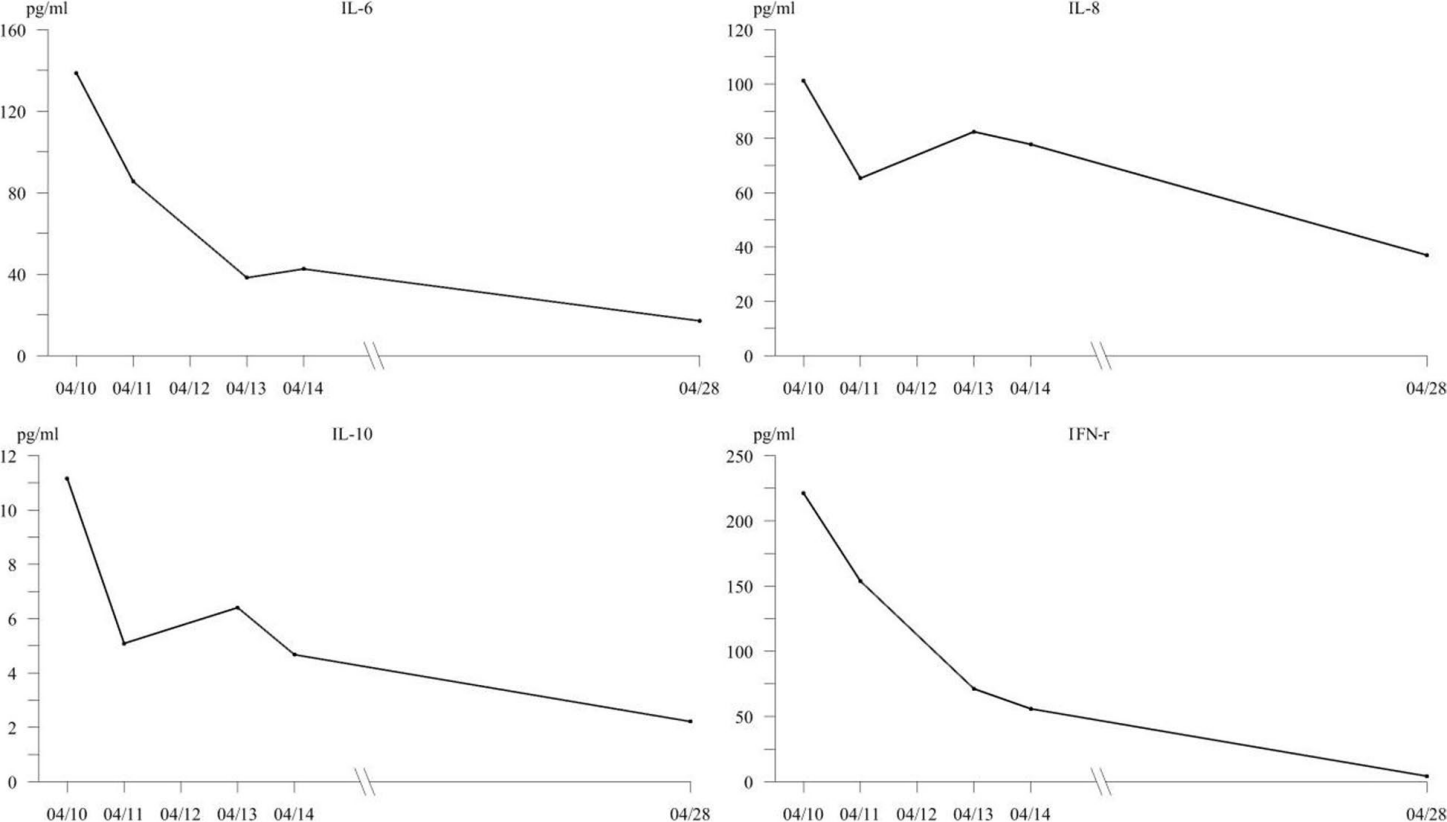
The trends of the averages of IL-6, IL-8, IL-10 and IFN-r in 9 of the imported acute histoplasmosis patients (except the No.1 patient). The normal reference ranges are: IL-6 < 5.40 pg/ml, IL-8 < 20.60 pg/ml, IL-10 < 12.90 pg/ml, and INF-γ < 3.93 pg/ml

Results showed that:

1. As the No.1 patient had the worst disease condition, the cytokines/chemokines (except for IL-4) of No.1 patient were much higher;
2. IL-4 was not detected in all patients (< 1.3 pg/ml);
3. IL-12, INF-α, TNF-α were not detected in 9 patients (No.2-No.10) (IL-12 < 1.76 pg/ml, INF-α < 1.05 pg/ml, TNF-α < 1.94 pg/ml);
4. most cytokines/chemokines (other than IL-4, IL-12, INF-α, TNF-α) varied widely with patients and time;.
5. values of the factors of No.1 patient fluctuated greatly, but all returned close to the normal ranges after 3 weeks’ treatment. For the other 9 patients, except for IL-4, IL-12, INF-α, TNF-α, the other factors all had an consistent overall trend, showing highest at the admission, decreasing rapidly within the first 4 days of hospitalization, and decreasing gradually after 4 days. This trend holds especially for the IL-6, IL-8, IL-10 and IFN-γ (Fig. 4).

### Spearman’s rank correlation of the markers and cytokines/chemokines

Rank correlations suggest that: 1) the following markers are in moderate positive associations to CRP and PCT: β2-MG, CysC, G-test, N/L, IL-6 and IL-27; 2) LDH and IL-10 are in weak positive associations to CRP, but in moderate positive associations to PCT; 3) IL-8 is in moderate positive association to CRP, but in weak positive association to PCT. More details are listed in Table 3 and Table 4.

**Table 3:** Spearman’s rank correlations between any two of the laboratory indicators

	CRP		PCT		G-test		CD4/CD8		WBC		N/L		β2-MG		Cysc		LDH	
	mean	median	mean	median	mean	median	mean	median	mean	median	mean	median	mean	median	mean	median	mean	median
CRP			0.49	0.53	0.46	0.47	−0.27	− 0.13	0.11	0.02	0.50	0.54	0.49	0.56	0.47	0.52	0.28	0.28
PCT					0.57	0.52	−0.08	− 0.08	0.08	0.04	0.48	0.50	0.61	0.58	0.46	0.38	0.53	0.50
G-test							−0.41	− 0.41	− 0.02	− 0.12	0.43	0.42	0.16	0.19	0.32	0.21	0.46	0.45
CD4/CD8									0.18	0.13	−0.02	0.08	0.18	0.18	− 0.17	− 0.17	− 0.55	− 0.55
WBC											0.37	0.47	0.15	0.13	0.24	0.18	−0.04	− 0.04
N/L													0.42	0.43	0.53	0.58	0.09	0.02
β2-MG															0.75	0.78	0.24	0.19
Cysc																	0.29	0.25

**Table 4:** Spearman’s rank correlations between cytokines/chemokines and the laboratory indicators

	CRP		PCT		G-test		CD4/CD8		WBC		N/L		PLT		β2-MG		Cysc		LDH	
	mean	median	mean	median	mean	median	mean	median	mean	median	mean	median	mean	median	mean	median	mean	median	mean	median
1 L-1β	0.00	0.15	0.25	0.20	0.23	0.24	−0.39	−0.39	0.04	0.05	0.16	0.05	− 0.01	−0.07	0.26	0.38	0.26	0.21	0.21	0.25
IL-2	0.30	0.24	0.17	0.20	0.15	0.12	−0.68	−0.68	0.00	0.05	0.05	0.07	−0.46	−0.59	0.23	0.26	0.25	0.31	0.49	0.61
IL-5	0.24	0.22	−0.06	−0.16	0.00	0.08	−0.45	−0.45	0.29	0.25	−0.10	−0.28	0.08	0.02	0.14	0.12	0.36	0.39	0.26	0.15
IL-6	0.53	0.59	0.54	0.53	0.38	0.31	−0.61	−0.61	0.08	−0.08	0.39	0.48	−0.43	−0.50	0.38	0.42	0.34	0.42	0.52	0.54
IL-8	0.50	0.62	0.22	0.37	0.21	0.22	−0.43	−0.43	− 0.12	0.15	0.13	0.20	−0.24	−0.37	0.18	0.16	0.17	0.22	0.55	0.61
IL-10	0.37	0.24	0.60	0.68	0.44	0.64	−0.55	−0.55	− 0.19	−0.21	0.23	0.19	−0.32	−0.43	0.42	0.37	0.16	0.14	0.58	0.60
IL-17	0.25	0.25	0.20	0.24	0.01	0.04	−0.41	−0.41	0.02	−0.18	0.13	0.15	−0.30	−0.50	0.27	0.24	0.08	0.18	0.22	0.16
IL-27	0.60	0.68	0.47	0.48	0.51	0.38	−0.52	−0.52	− 0.35	−0.22	0.30	0.33	−0.50	−0.59	0.16	0.16	−0.06	−0.03	0.50	0.56
IL-33	0.21	0.31	0.24	0.47	0.15	0.30	−0.30	−0.30	−0.04	− 0.13	0.20	0.32	−0.21	− 0.41	0.28	0.41	0.15	0.19	0.19	0.24
IFN-r	0.37	0.37	0.19	0.20	0.21	0.21	−0.64	−0.64	0.04	0.14	0.18	0.22	−0.36	−0.42	0.34	0.28	0.29	0.24	0.22	0.10
HMGB1	0.10	0.21	0.30	0.31	0.15	0.22	0.01	0.01	0.09	0.10	0.07	0.03	−0.20	−0.22	0.10	0.25	−0.09	0.04	0.12	0.26

## Discussion

Acute histoplasmosis is a rare fungal disease. Staffolani et al. [12] once reviewed the literatures on acute histoplasmosis in immunocompetent travelers. They found most reported travelers were from America and Europe to Africa and South America. The only exception they found was traveling from Taiwan to Indonesia. By then, no immunocompetent travelers from Chinese mainland was found by them. In recent years, an increasing number of sporadic and chronic histoplasmosis cases occur in Chinese mainland, 90–95% of which are asymptomatic in immunocompetent persons.

The infection of acute histoplasmosis is usually mild and self-limited in immunocompetent people, however, in rare cases, the disease can progress to severe form with high morbidity [13–16]. The most frequent symptoms are fever, cough, headache and chest pains. Constitutional symptoms (myalgia, sweats, weight loss, anorexia etc.) are also commonly reported. All 10 patients in this study had accordant clinical symptoms with those in literatures, but the condition of these patients especially No.1 patient was apparently much worse than those in literatures [2, 17–27]. This is possibly because China is a non-endemic country and most Chinese are unimmune to acute histoplasmosis, Moreover, those patients did not take any respiratory protection when they were exposed for long time inside the tunnel, as a result they had to inhaled a large amount of pathogen in these days. In addition, due to the limitation of local healthcare facilities, they did not receive timely diagnosis and treatment, as a result they were already very seriously infected when they were back in China, already 8–18 days after the onset.

The most common sign of chest CT imaging in literatures is nodular infiltrates [28]. In this study, CT images in chests of patient No.1-No.4 showed diffuse miliary nodules in both lungs, while the images of patient No.5-No.10 showed only scattered nodular shadows. This difference is probably related to the difference in their jobs, and accordingly in their exposure conditions. Patients No.1-No.4 were soil cleaners who underwent longer exposure and inhaled more pathogenic fungi, consequently, their disease conditions were much worse than the others. Thus obviously not only the pulmonary nodules, but also the severity and manifestations of the acute histoplasmosis, are closely related to the inhaled fungi amount and the exposure time.

The abdominal CT manifestations in literatures include splenomegaly (sometimes with focal splenic lesions) and lymph node enlargement [29]. Although diffuse hypodense lesions are less common in spleen, they are regarded as specific signs of histoplasmosis infection [30]. As none of imaging manifestations of hepatosplenomegaly was found previously in these patients, the reason for the current hepatosplenomegaly of the patients maybe relevant to the spread of histoplasm along the reticuloendothelial system after respiratory infection. But we did not take further histological examination of liver and spleen for the verification, because of the disagreement of the patients.

It’s reported 5–20% of disseminated histoplasmosis can affect the central nervous system, especially in immunosuppressive patients [31], or sometimes in patient with normal immunity [32]. In this study, the No.1 patient was firstly in drowsiness and then in coma all the time. Although his cerebrospinal fluid was cultured and was negative, unfortunately no further relevant examination was allowed by his families at that time, thus we can hardly exclude the possibility of the spreading of histoplasm over the central nervous system. It is possible that not only the brain parenchyma but also the meninges had been damaged when the histoplasm spread into the nervous system.

From the laboratory test results of the 10 patients, we could find the ratio of CD4/CD8 was < 1, and the CRP, PCT, G-test, LDH, CysC, β2-MG were mostly elevated by varying degrees. However, none of them showed specificity in the diagnosis of acute histoplasmosis. However, we must note that, microbiological diagnosis methods (e.g. culture), and histopathology are still unsubstitutable. The gold standards for the diagnosis of histoplasmosis are still direct microscopic examination and culture. In this study, even the result of mNGS test was also taken as a reference, while the final definitive diagnosis was still based on the fungi growth in cultures, as the sensitivity of mNGS test was reported to possibly vary widely from 36 to 100% [33]. For infections with unknown pathogens, detailed investigation of epidemiological history is very important. The patients in this study were all infected when they were working in an endemic area of histoplasmosis, As the 10 patients had the similar clinic symptoms, and also the results of their imaging examination, blood laboratory test and blood mNGS test all hinted the existence of H. capsulatum, so all of them were considered as acute histoplasmosis patients and were treated accordingly. The final microbiological evidence also verified this diagnosis.

The CRP and PCT are inflammation indicators showing more specificity to the acute infections, not only bacterial but also fungal [34, 35]. The G-test is often employed in the diagnosis of fungal infection, and it should be gradually decreased by anti-infective therapy [36, 37], because the fungal (1–3)-β-Dglucan is a primary composition of fungal cytoderm and is released via hydrolyzation during fungal infection [38]. Thus it might be reasonable that, a decrease of fungal (1–3)-β-Dglucan along with the anti-infective therapy possibly indicates a fungal infection [37]. In this study, according to the variation of disease conditions of the patients, the CRP, PCT and the G-test all show consistent trends with the disease conditions that, they were all high at the admission but significantly decreased 3 weeks later when disease conditions of patients turned better. Besides, with regard to other factors, we can find 1) the CysC, β2-MG, LDH also show very consistent trends with the PCT and CRP, and find 2) the rank correlation coefficients between CysC, N/L, β2-MG, LDH and G-test, CRP, PCT are all positive. In this case, although it is not solidly suggested, it can to some extent be indicated that the CysC, N/L, β2-MG, LDH can reflect the possibility of airway inflammation, and have certain with the disease condition. We’d suggest that it worths trying to include those factors in the condition monitoring of acute histoplasmosis patients, which might also help in the prognosis and follow up.

Cytokines, particularly chemokines, are key recruitment mediators of leukocytes and other inflammatory cells leading to pulmonary damage. They have been implicated in the pathogenesis of ARDS/diffuse alveolar damage [39]. Now the monitoring of cytokines/hemokines is commonly applied in sepsis [40], severe acute respiratory syndrome (SARS) [41] and 2019 novel coronavirus infection disease (COVID-19) [42]. But there are not many studies about the monitoring and correlation analysis of cytokines/chemokines in acute histoplasmosis patients. In this study, we measured 15 cytokines/chemokines in 10 patients during hospitalization. Perhaps this is the first study on continuous monitoring of cytokines/chemokines in acute histoplasmosis.

For the No.1 patient, all cytokines/chemokines other than IL-4 were elevated more highly (around 10 times) than other patients, which may be due to his serious illness and the septic shock, ARDS and cardiopulmonary resuscitation he once experienced in the early stage. In the following treatment course those factors fluctuated, but after 3 weeks’ treatment, those factors returned close to normal, most of his symptoms disappeared, and the infection of his lungs remitted as well. The other 9 patients’ most cytokines/chemokines (other than IL-4, IL-12, INF-α, TNF-α) varied widely with patients and time. The general trend of those factors is highest at admission, decreasing rapidly in the first 4 days of hospitalization, and decreasing gradually thereafter. This trend holds especially for the IL-6, IL-8, IL-10 and IFN-γ. In addition, the IL-6, IL-8, IL-10 and IFN-γ all show positive rank correlation coefficients with the CRP and PCT, thus it could be kind of indication for that the IL-6, IL-8, IL-10 and IFN-γ can somewhat reflect the condition of airway inflammation, and may be relevant with the prognosis as well. Although IL-27 has moderate associations to CRP and PCT as well, it’s not taken into this discussion because it was mostly within normal range.

Despite some characteristics of those laboratory test factors in acute histoplasmosis patients were found in this study, due to the limitation of the number of samples, and due to the special condition of patients, our study is mainly a qualitative observational study. In this case, the results and conclusions of this study still require further verification by studies with more samples and with randomized controlled trials, or by in vitro cell experiments and animal experiments.

## Supplementary Information

**Additional file 1: Supplement table 1.** The arterial blood gas analysis in the 10 patients at admission.

## Data Availability

All data is included in this published article. Further inquiries can be directed to the corresponding author.

## Abbreviations

H: capsulatum Histoplasma capsulatum
PCR: Polymerase chain reaction
mNGS: Metagenomic Next-generation Sequencing
CT: Computer tomography
CRP: C-reactive protein
LDH: Lactate dehydrogenase
BALF: Bronchoalveolar lavage fluid
HE: Hematoxylin-Eosin
PAS: Periodic Acid-Schiff
PCT: Procalcitonin
G-test: fungal (1–3)-β-Dglucan G-test
EDTA: Ethylenediamine tetraacetic acid
HMGB1: High mobility group protein-1
WBC: White blood cell count
N/L: the ratio of neutrophils to lymphocytes
β2-MG: β2-microglobin
CysC: Cystatin C
ELISA: Enzyme-linked immunosorbent assay\
PCR: Polymerase chain reaction
ARDS: Acute respiratory distress syndrome
SARS: Severe Acute Respiratory Syndrome (SARS)
COVID-19: 2019 novel coronavirus infection disease
