# Supplementary material for: Collective outbreak of severe acute histoplasmosis in immunocompetent Chinese in South America: the clinical characteristics and continuous monitoring of serum cytokines/chemokines
Source: BMC Prim Care. 2022 Aug 8;23:197. doi: 10.1186/s12875-022-01771-2 (PMC9358111; doi:10.1186/s12875-022-01771-2)
Supplement: Supplementary file 1 — Additional file 1: Supplement table 1. The arterial blood gas analysis in the 10 patients at admission. [file 12875_2022_1771_MOESM1_ESM.docx]

supplement table 1: The arterial blood gas analysis in the 10 patients at admission.

| patient No. | 1 | 2 | 3 | 4 | 5 | 6 | 7 | 8 | 9 | 10 |
| --- | --- | --- | --- | --- | --- | --- | --- | --- | --- | --- |
| PO_2_ (mm Hg) | 109 | 50 | 70 | 88 | 67 | 111 | 90 | 74 | 83 | 76 |
| PCO_2_ (mm Hg) | 41 | 34 | 30 | 40 | 43 | 35 | 40 | 42 | 40 | 39 |
| FiO_2_ (%) | 100 | 41 | 33 | 33 | 33 | 33 | 33 | 33 | 33 | 33 |
| PO_2_/FiO_2_ | 109 | 122 | 212 | 267 | 203 | 336 | 273 | 224 | 252 | 230 |
| SPO_2_ (%) | 99 | 85 | 96 | 97 | 93 | 98 | 97 | 95 | 96 | 96 |

Note: The No.1 patient was assisted by invasive mechanical ventilator, and the others all used nasal cannulas and face masks for oxygen.
